# The relationship between buildings and health: a systematic review

**DOI:** 10.1093/pubmed/fdy138

**Published:** 2018-08-18

**Authors:** Janet Ige, Paul Pilkington, Judy Orme, Ben Williams, Emily Prestwood, D Black, Laurence Carmichael, Gabriel Scally

**Affiliations:** 1 Department of Health and Social Sciences, University of the West of England, Bristol, UK; 2 Air Quality Management Resource Centre, University of the West of England, Bristol, UK; 3 Daniel Black + Associates | db+a, Bristol, UK; 4 WHO Collaborating Centre for Healthy Urban Environments, University of the West of England, Bristol, UK

**Keywords:** buildings, health, housing

## Abstract

**Background:**

The built environment exerts one of the strongest directly measurable effects on physical and mental health, yet the evidence base underpinning the design of healthy urban planning is not fully developed.

**Method:**

This study provides a systematic review of quantitative studies assessing the impact of buildings on health. In total, 7127 studies were identified from a structured search of eight databases combined with manual searching for grey literature. Only quantitative studies conducted between January 2000 and November 2016 were eligible for inclusion. Studies were assessed using the quality assessment tool for quantitative studies.

**Results:**

In total, 39 studies were included in this review. Findings showed consistently that housing refurbishment and modifications, provision of adequate heating, improvements to ventilation and water supply were associated with improved respiratory outcomes, quality of life and mental health. Prioritization of housing for vulnerable groups led to improved wellbeing. However, the quality of the underpinning evidence and lack of methodological rigour in most of the studies makes it difficult to draw causal links.

**Conclusion:**

This review identified evidence to demonstrate the strong association between certain features of housing and wellbeing such as adequate heating and ventilation. Our findings highlight the need for strengthening of the evidence base in order for meaningful conclusions to be drawn.

## Introduction

Although the relationship between the built environment and health is complex, the effect of buildings on health, particularly housing, has been recognized for over a century.^1^ The indoor environment is quite integral to wellbeing. People spend most of their time indoors, at home or work;^2^ yet in a developed country such as the UK, 4.6 million homes (19% of the total) failed to meet the decent home standard in 2015.^3^ This standard identifies that a ‘decent home’ is in a reasonable state of repair, has reasonable modern facilities and services, and provides a reasonable degree of thermal comfort.^3^

Research on the impact of design and quality of buildings on health and wellbeing of occupants has been widely reported,^4^ though there are substantial gaps in the evidence. The risk of asthma and other respiratory conditions have been shown to increase among children living in damp houses,^5^ whilst the accessibility of buildings has become an increasingly important consideration for older adults^6^—a demographic which spends a higher proportion of time in their homes and neighbourhood than any other age group.^7^ A consequence of poor housing quality is the associated health cost. In the UK for instance, the Building Research Establishment estimates that the NHS spends about £600 million per annum on direct health costs associated with attending to hazards in the worst housing stock in England^8^ while the National Housing Federation asserts that about £2.5 billion per annum is spent in attending to housing and health-related conditions across the UK.^9^ In addition to the impact of housing quality on health, there is some evidence to suggest that other types of buildings, including offices and school buildings, can affect health and wellbeing.^10^ Active design within buildings and access to amenities has been shown to improve active living and increase productivity,^11^ though this area is currently under-researched.

A number of studies have investigated the association between specific features of housing and health outcomes.^12,13^ However, there is insufficient systematic review level evidence to provide a comprehensive picture of how several features of building design affect health and wellbeing at the population level. A review study investigating the effects of housing improvement on health and wellbeing reported inconclusive findings due to the lack of evidence.^14^ A follow-up review in 2009 identified associations between energy efficiency and respiratory health, but the findings were limited in the extent to which conclusions could be drawn on the impact on health inequalities.^15^ Another follow-up Cochrane review by the same authors investigated the socioeconomic impact of housing improvements and identified significant evidential gaps in relation to the impact of housing improvement on social and economic outcomes.^16^

The true magnitude of the impact of buildings on health and wellbeing cannot be fully understood without a systematic synthesis and quality assessment of the literature reporting these associations. In addition, the identification and collation of scientifically robust and credible evidence, based on existing literature is necessary to produce a structured evidence base that can resonate with and support policy makers and experts in the built environment arena. This study therefore aims to systematically review the impact of buildings on health. In addition to presenting these findings to aid urban development decision makers, the study also provides the basis for a next phase economic evaluation of the impact of building quality on health and wellbeing.

## Method

### Search strategy

A list of potentially relevant databases was compiled from existing systematic reviews across similar topics^16–18^ and in consultation with experts in the field. Eight electronic databases (MEDLINE, PsychINFO, Cumulative Index to Nursing and Allied Health Literature, Applied Social Sciences Index and Abstracts, Cochrane Database of Systematic Reviews, SocINDEX, EconLit, Allied and Complementary Medicine) were searched by heading and abstract to identify relevant publications from January 2000 to November 2016.

The search terms were categorized into three-word groups relating to characteristic of the built form, study type and health outcomes (Appendix 1). Following an initial draft of search terms, subject area experts were contacted to verify and refine the terms. A pilot search was performed by the project researcher (J.I.) in one database (MEDLINE) to test the search strategy and refine the search terms before the full search was undertaken by the same researcher. Additional searches were conducted by JI and DB on Google or Google Scholar to locate potentially eligible studies and grey literature. All authors were involved in identifying relevant grey literature. This was combined with manual searching of referenced articles by J.I. Two reviewers (J.I. and P.P.) independently assessed the quality of selected studies and extracted relevant data. The reporting of this review conforms to recommendations from the Preferred Reporting Items for Systematic Reviews and Meta-Analyses (PRISMA).^19^

### Eligibility

To be selected for inclusion, studies were required to meet the following inclusion criteria: (i) report on quantifiable associations between any building related variable and health outcomes (primary or secondary); (ii) be published in English language between January 2000 to November 2016 with full text in a peer-reviewed journal or nationally recognized stakeholder website. (The limit on year of publication is in order to reflect the contemporary issues in building design.) (iii) Be conducted in a high income country according to the World Bank categorization.

Qualitative studies were excluded from this review as the main aim of the study was to identify the quantifiable impact of the built environment on health. In line with previous systematic reviews,^20,21^ the quality assessment tool for quantitative studies, developed by the Effective Public Health Practice Project (EPHPP) was used to rate the quality of included studies. This tool was selected for its ability to assess methodological rigour across a range of observational and empirical studies. The tool has been recommended for rating the methodological quality of studies based on construct validity and acceptable content.^22,23^ The tool consists of six quality assessment domains: (i) The probability that the study participants are representative of the target group (selection bias); (ii) design of the study; (iii) the control of confounding factor; (iv) the concealment of participants and researchers (blinding); (v) the reliability and validity of data collection methods; and (vi) reporting of withdrawals and dropout rate.^22,24,25^ These individual components were rated high, moderate or low.

## Results

A total of 39 studies met the eligibility criteria and were included in the review. Of these, 15 were conducted in the UK, 13 were from USA, 3 were from New Zealand and the remaining 8 studies were from Australia, Canada South Korea and the rest of Europe.

The majority of the identified literature reporting on the links between building design and health was cross-sectional, with a small sample size that limits the generalizability of findings.^26–30^ About 62% of the studies (*n* = 23) included in the final selection were limited by poor study design and weak methodological rigour and hence excluded from synthesis. The studies included in the synthesis comprised of 4 studies of high quality and 12 studies of moderate quality.

**Fig. 1:**
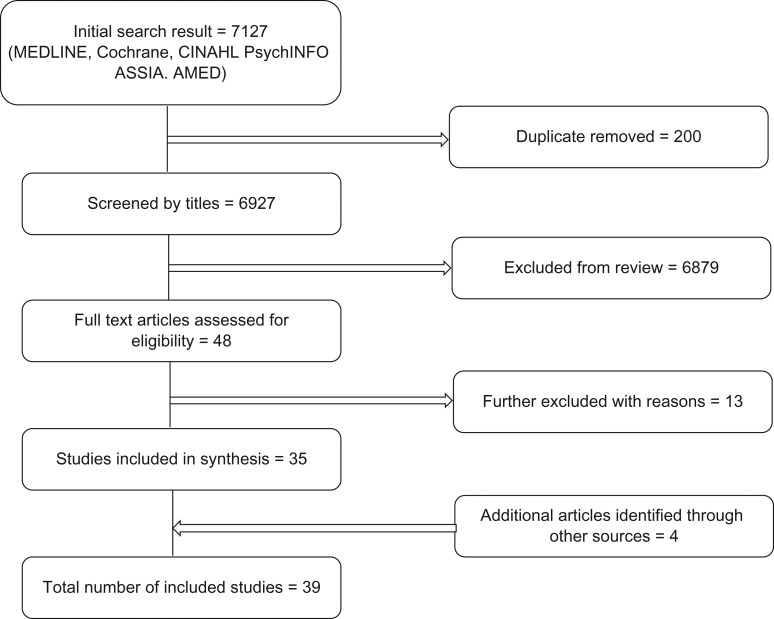
Study selection process.

**Fig. 2:**
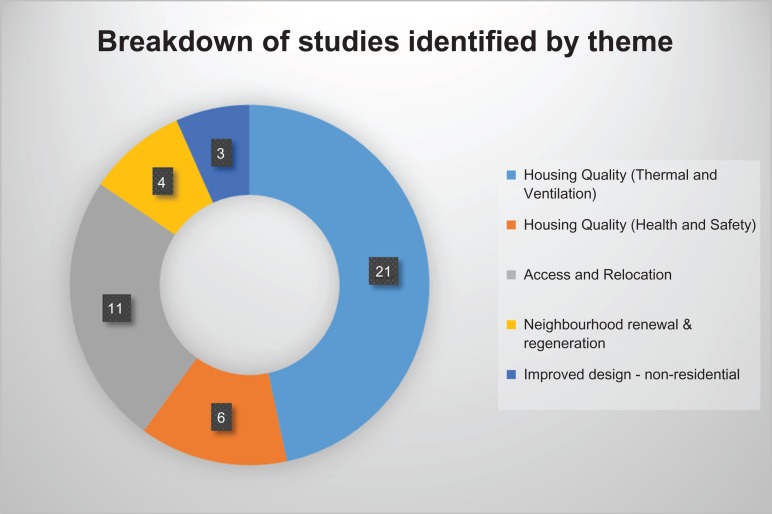
Key themes emerging from included studies.

The result of the search is summarized in Figs 1 and 2 and the summary of findings are presented in Appendices 2 and 3.

### Review findings

#### Quality of housing (thermal and ventilation)

Seven studies investigated the health impact of interventions to improve ventilation and warmth in residential housing. Of these, three randomized control trials (RCTs),^31–33^ examined the impact of central heating and ventilation on health and wellbeing of children. Both interventions were associated with significant improvements in self-reported/parent-reported respiratory outcomes and general wellbeing across all three studies. In addition, housing warmth was positively associated with school attendance across all three studies. However, this association was only statistically significant in one of the studies.^31^ Another study^32^ reported positive effects of replacement of unfuelled gas heaters with fuelled gas heaters, heat pump or wood pellet heaters on reduction of nitrogen dioxide (NO_2_) levels in living rooms. These studies are set out in more detail in Table 1.

**Table 1:** Main findings from studies on thermal quality and ventilation

Study, location	Study design^a^	Aim(s)	Main findings	Quality of study
Aylin *et al.*,^38^ UK	Ecological study	To evaluate the associations between temperature, housing, deprivation and excess winter mortality	There was a significant association between excess winter mortality and temperature. For every 1°C reduction in 24 h mean winter temperature there was a 1.5% increased odd of dying. Associations between housing and winter mortality were not statistically significant; however, lack of central heating was associated with higher risk of dying in winter (OR = 1.016, 95% CI = 1.009–1.022).	Moderate
Curl *et al.*,^37^ UK	Q	To evaluate the impact of housing improvements on physical and mental health	Fabric works (which includes over-cladding and insulation) showed positive associations with physical health (+2.09, 95% CI = 0.13–4.04) and mental health (+1.84, 95% CI = 0.04–3.65) in 1–2 years. Improvements to kitchens and bathrooms demonstrated a positive association with mental health in 1–2 years (+2.58, 95% CI = 0.79–4.36). Central heating had a negative association with physical health (−2.21, 95% CI = −3.74 to −0.68). New front doors had a positive association with mental health in <1 year (+5.89, 95% CI = 0.65–11.14) and when provided alongside kitchens and bathrooms (+4.25, 95% CI = 1.71–6.80).	Moderate
Edward *et al.*,^34^ UK	CEA nested in RCT	To evaluate the cost-effectiveness of installing ventilation systems in homes of children with moderate to severe asthma	The intervention (described in Woodfine *et al.*^33^) was successful in shifting 17% of children with severe asthma to moderate asthma, compared with a 3% shift in the control group. The mean cost of the intervention was £1718 per child treated or £12 300 per child shifted from severe to moderate. An incremental cost efficiency ratio (ICER) of £234 was obtained per point improvement on the 100-point asthma scale (PedsQL). 95% Confidence interval (CI) = £140–590. ICER declined to £165 (95% CI = £84–424) for children with ‘severe’ asthma.	Moderate
Howden-Chapman *et al.*,^32^ New Zealand	RCT	To examine the effect of improved home heating on asthma among children	The intervention group were provided with non-polluting, more effective home heating before winter, while the control group received replacement heater at the end of the trial. There was no significant difference in improvement in lung function among intervention and control group at the end of 1 year. However, children in the intervention group had 1.80 fewer days off school (95% CI = 0.11–3.13), 0.40 fewer visits to doctor for asthma (95% CI = 0.11–0.62), and 0.25 fewer visits to a pharmacist for asthma (0.09–0.32). Children in the intervention group also had fewer reports of poor health (adjusted odds ratio = 0.48, 95% CI = 0.31–0.74), less sleep disturbed by wheezing (0.55, 0.35–0.85), less dry cough at night (0.52, 0.32–0.83), and reduced scores for lower respiratory tract symptoms (0.77, 0.73–0.81) than children in the control group. The intervention was associated with a mean temperature rise in the living room of 1.10°C (95% CI: 0.54–1.64°C) and in the child’s bedroom of 0.57°C (0.05–1.08°C). Lower levels of nitrogen dioxide were measured in the living rooms of the intervention households than in those of the control households (geometric mean 8.5 μg/m^3^ versus 15.7 μg/m^3^, *P* < 0.001). A similar effect was found in the children’s bedrooms (7.3 μg/m^3^ versus 10.9 μg/m^3^, *P* < 0.001).	Moderate
Woodfine *et al.*,^33^ UK	RCT	To evaluate the effectiveness of installing ventilation systems in the homes of children with moderate or severe asthma	The intervention improved parent-reported asthma specific quality of life significantly at both 4 and 12 months. The adjusted mean difference for the PedsQL asthma summary score of the two groups at 12 months = 7.1 points (95% CI = 2.8–11.4, *P* = 0.001; standardized effect size = 0.42). The generic quality-of-life scale showed that health problems were significantly reduced at 4months (adjusted mean difference of 7.2, 95% CI = 2.6–11.8, *P* = 0.002), while result were not significant at 12 months (mean difference = 4.5, 95% CI = –0.2 to 9.1, *P* = 0.061). School attendance was higher in the intervention group, albeit this was not statistically significant (Mann–Whitney *U* tests: *P* = 0.091 for all-cause absence, *P* = 0.053 for asthma-related absence).	Moderate
Barton *et al.*,^35^ UK	RCT	To assess the short-term (1 year period) health effects of housing improvement	Houses and residents were randomized to two groups: group 1 received upgrade to ventilation, heating, insulation and other home improvements in the first year (intervention group) and group 2 were on a waiting list to receive the same kind of upgrade in the second year (control group). A postal questionnaire was sent to residents; outcomes were measured using annual health questionnaires SF36 and GHQ12. All adults were later interviewed by a trained community nurse. The interventions (central heating, ventilation, rewiring, insulation and re-roofing) improved energy efficiency. Residents of un-improved houses (control group) reported increase in non-asthma-related chest problems including bronchitis, dry throat, itchy eyes, blocked nose and runny nose (Mann–Whitney test, *z* = 2.8; *P* = 0.05). Adults in improved houses showed improvement in combined asthma symptom score (Mann–Whitney test, *Z* = 2.7; *P* = 0.07). No difference was observed between the intervention and control group for SF36 or GHQ12 (tools for measuring general health).	High
Dedman *et al.*,^36^ UK	Cohort	To examine the association between measures of housing condition during childhood and all-cause mortality	Inadequate housing conditions were generally associated with increased adult mortality. After adjusting for childhood and adult socioeconomic factors, indoor tapped water supply was significantly associated with increased mortality from coronary heart disease (hazard ratio = 1.73, 95% CI = 1.13, 2.64); Similarly, significant association was observed between poor ventilation and overall mortality (hazard ratio for people from households with poorest ventilation relative to best ventilation 1.30, 95% CI = 0.97, 1.74).	High
Howden-Chapman *et al.*,^31^ New Zealand	Cluster RCT	To examine whether insulating existing houses can increase indoor temperature and improve occupants’ health and wellbeing	Intervention group reported a slight increase in bedroom temperatures during the winter (0.5°C) and decrease in relative humidity (−2.3%). However, energy consumption in insulated houses was 81% of that in uninsulated houses. These changes were significantly associated with reduced odds of fair or poor self-rated health (adjusted odds ratio = 0.50, 95% CI = 0.38–0.68), self-reports of wheezing in the past 3 months (OR = 0.57, 0.47–0.70), self-reports of children taking a day off school (0.49, 0.31–0.80), and self-reports of adults taking a day off work (0.62, 0.46–0.83). The odds of hospital visits were lower among occupants of insulated homes (0.73, 0.62–0.87). Hospital admissions for respiratory conditions were also reduced (0.53, 0.22–1.29), but this reduction was not statistically significant (*P* = 0.16).	High

A cost-effectiveness study examined the cost-savings from improving ventilation and installing central heating on health and wellbeing of children with asthma (Tables 1 and 2).^34^ The authors reported an incremental cost efficiency ratio (ICER) of £234 per point improvement on the 100-point asthma scale (95% confidence interval (CI) = £140–590). The probability of the intervention to be cost effective was 97.5% at £590.

**Table 2:** Main findings from studies on health and safety of housing

Study, location	Study design^a^	Aim(s)	Main findings	Quality of study
Blackman *et al.*,^41^ UK	B-A	To investigate the association between housing renewal (fabric repairs) and health	After controlling for confounding variables, findings showed that an adult living in a damp house is significantly more likely to have one or more acute respiratory condition (OR = 2.1, 95% CI = 1.26–3.5). Findings also show that perception of an area as being unsafe can increase odds of mental health problems (adjusted OR = 2.35, 95% CI = 1.41–3.92). An adult living in a dwelling with serious drought is significantly more likely to report a mental health problem than one living in a dwelling with minor or no drought (OR = 2.28, 95% CI = 1.41–3.69). In terms of children, living in a damp house increases odds of one or more respiratory problems by 3.5 (95% CI = 1.69–7.18).	Moderate
Curl *et al.*,^37^ UK	Q	To evaluate the impact of housing improvements on physical and mental health	Fabric works (which includes over-cladding and insulation) showed positive associations with physical health (+2.09, 95% CI = 0.13–4.04) and mental health (+1.84, 95% CI = 0.04–3.65) in 1–2 years. Improvements to kitchens and bathrooms demonstrated a positive association with mental health in 1–2 years (+2.58, 95% CI 0.79 to 4.36). Central heating had a negative association with physical health (−2.21, 95% CI = −3.74 to −0.68). New front doors had a positive association with mental health in < 1 year (+5.89, 95% CI = 0.65–11.14) and when provided alongside kitchens and bathrooms (+4.25, 95% CI = 1.71–6.80).	Moderate
Vettore *et al.*,^40^ Brazil	Case-C	To examine the relationship between housing condition and low birthweight and preterm low birthweight among low-income women.	Housing conditions were grouped into three categories: adequate, inadequate and highly inadequate. Findings show that poor housing conditions was independently associated with low birthweight (inadequate-adjusted OR = 2.2 , CI = 1.1–4.3 highly inadequate-adjusted OR = 7.6, CI = 2.4–23.9).	Moderate
Dedman *et al.*,^36^ UK	Cohort	To examine the association between measures of housing condition during childhood and all-cause mortality	Inadequate housing conditions were generally associated with increased adult mortality. After adjusting for childhood and adult socioeconomic factors, of private indoor tapped water supply was significantly associated with increased mortality from coronary heart disease (hazard ratio 1.73, 95% CI = 1.13, 2.64); Similarly, significant association was observed between poor ventilation and overall mortality (hazard ratio for people from households with poorest ventilation relative to best ventilation 1.30, 95% CI = 0.97, 1.74).	High
Keall *et al.*,^39^ New Zealand	Clustered RCT	To assess the safety benefit of home modifications	Households were randomly assigned into immediate home- modification (intervention group) or a 3-year wait before modification (control group). Findings show that following 1148 days of randomization, the crude rate of fall injuries per person per year in the intervention and control group was 0.061 and 0.072, respectively. In addition, the crude rate of injuries specific to home modification intervention was 0.018 in the intervention group and 0.028 in the control group. After adjusting for relevant confounders, there was a 26% reduction in the rate of home injuries caused by falls in the group that received home modification (relative risk = 0.74, CI = 0.058–0.94). Injuries specific to the intervention also declined by 39% per year among those that received the intervention (RR = 0.61, CI = 0.41–0.91).	High

Four studies including two RCTs, one cohort study and one quasi-experimental study investigated the effect of central heating, improving ventilation and insulation on health and wellbeing of adults (Table 1). Of these, three studies identified positive effects of ventilation and central heating on respiratory outcomes and physical health.^31,35,36^ Only one study found an adverse association between central heating and physical health at twelve months (as measured by the Physical Health Composite Scale). This effect was however outweighed by significant improvements in mental health at three years (measured by the Mental Health Composite Scale).^37^

Findings from an ecological study that examined the associations between temperature, housing deprivation and excess winter mortality showed that lack of central heating could significantly increase the risk of excess winter deaths.^38^

#### Quality of housing (health and safety)

Five studies investigated the impact of housing conditions on health and safety of occupants.^36,37,39–41^ Three of these studies assessed the health outcomes associated with housing renewal, modification/improvement and two of them reported positive correlation with falls prevention^33^ and health improvement.^37^ Housing modification refers to structurally affixed modifications to the home to enable independent living. This could include ramps, rails, lighting improvements, level access showers. Blackman *et al.*^41^ found that perception of an area as unsafe could negatively affect mental health while presence of damp in the house increases the odds of acute respiratory illness, with children reporting higher odds than adults.

Another study^40^ found that pregnant women living in ‘highly inadequate’ housing, described as non-urbanized areas without sewage systems, were ~7.6 times more likely to have babies with low birthweight compared to those living in ‘adequate housing’. A cohort study^36^ reported that children living in poor housing conditions had poorer health outcomes and higher odds of mortality in adulthood (Table 2). The markers used for identifying and categorizing housing conditions were: overcrowding, water supply, toilet facilities, adequacy of ventilation and cleanliness of households.

#### Housing affordability/access to affordable homes or social housing

Five studies assessed the effectiveness of access/relocation to affordable homes.^42–46^ Two of the studies examined the benefit of immediate rental housing assistance to homeless people living with HIV/AIDS (PLWHA).^42,43^ The intervention was shown to reduce emergency department visits among the target population by 26% and resulted in a cost per Quality-Adjusted Life Year (QALY) savings of $6 249 3;^42^ this is above the recommended cost-effectiveness threshold by the National Institute for Health and Care Excellence in the UK.^47^

Two studies assessed the benefit of relocation from public housing in a neighbourhood of high poverty to private housing in low-poverty areas.^44,45^ Findings showed that relocation was associated with reduced depressive symptoms among adults and better educational achievement scores of boys aged 11–18 years (Table 3). The fifth study examined the effect of housing affordability on mental health among people from low-income groups and reported a slight decrease in mental health for those living in homes where the housing cost was more than 30% of their household income. This association was however not statistically significant.^46^

**Table 3:** Main findings from studies on housing affordability

Study, location	Study design^a^	Aim(s)	Main findings	Quality of study
Bentley *et al.*,^46^ Australia	L	To investigate the effect of housing affordability on mental health among people with low household income	This study was performed to evaluate the association between living in a house where the housing cost was more than 30% of household income and mental health. Data for the study were retrieved from an Australian National longitudinal survey. Mental health was measured using the self-completed Short Form SF36 measure. The authors found that entering unaffordable housing for individuals living in low-to-moderate income households was associated with a slight decrease in mental health score (mean change = −1.19, 96% CI = −1.97 to −0.41). There was no evidence for an association between mental health and affordable housing for higher income earners.	Moderate
Holtgrave *et al.*,^42^ USA	CUA of RCT study	Cost utility analysis of the impact of provision of immediate rental housing assistance to people living with HIV/AIDS	Cost Utility Analysis based on findings from a randomized controlled study to examine the impact of provision of immediate rental housing assistance to people living with HIV/AIDS (Housing and Health study described in Kidder, 2007). The cost per QALY saved by provision of rental housing assistance to homeless PLWHA was $62 493.	Moderate
Kidder *et al.*,^43^ USA	RCT	To assess the impact of provision of immediate rental housing assistance to people living with HIV/AIDS (PLWHA) who were homeless	A total of 630 participants completed baseline assessment and were randomized to either receive immediate rental housing assistance (intervention) or assistance with finding housing according to standard practice (Control). Findings demonstrate that health status of homeless people was poorer than that of housed respondents. Homeless respondents were also more likely to have visited an emergency department, and to have been admitted to a hospital. The 40% of homeless respondents (compared to 26% of housed respondents) were more likely to have visited emergency department (*P* < 0.001, *X*^2^ = 32.2). Relative to 21% of housed respondents, 37% of homeless participants were more likely to have been admitted in the hospital in the past 12 months (*P* < 0.001, *X*^2^ = 42.3). Homeless respondents had lower CD4 counts, were less likely to adhere to anti-retroviral therapy.	Moderate
Leventhal *et al.*,^44^ USA	RCT	To examine the short-term effects of relocation from public housing in neighbourhood of high poverty to private housing in low-poverty areas on mental health	Parents who moved to areas of low- poverty reported significantly less distress than counterparts who remained in areas of high poverty. Young boys who relocated to areas of lower poverty also reported significantly fewer anxiety issues than mates in public housing. There was a 20% reduction in depressive symptoms among experimental parents than control parents (*P* < 0.001).	Moderate
Leventhal and Brooks-Gunn,^45^ USA	RCT	To investigate the impact of relocation from public housing in neighbourhood of high poverty to private housing in low-poverty areas on children’s achievement, grade retention, suspensions and expulsions	Data from Leventhal 2003 was examined to access whether moving from high poverty neighbourhoods to low-poverty areas was associated with low-income minority children’s achievement, grade retention, suspensions and expulsions. Findings show that moving to low-poverty neighbourhoods had positive effects on 11–18-year-old boys’ achievement scores compared with those of their peers in high-poverty neighbourhoods.	Moderate

## Discussion

### Main findings of this study

Findings from this review strengthens the evidence base on the intricate association between building design and health outcomes. It provides an invaluable synthesis of the existing evidence, and a rigorous assessment of its quality—highlighting gaps for further focus as well as areas where the evidence is strongest.

The positive effects of housing warmth on respiratory health and wellbeing of children and adults was perhaps the most consistent and significant finding. Our findings also demonstrate the importance of providing affordable housing of good quality to vulnerable groups as a way of addressing the widening health inequality gap. Interventions to improve the quality of housing by maximizing energy efficiency, removing home hazards and adapting existing buildings were all associated with a number of positive health outcomes, including: improved quality of life, mental health and clinical health-related outcomes.

This review has also shown that there are significant gaps in the evidence in relation to non-residential buildings design and health. The three studies which assessed the quality of non-residential buildings were deemed to be of low quality and excluded from analysis.^48–50^ There were also substantial gaps in evidence, particularly on global, systems issues such as overheating in buildings and associated impact and outcomes.

The lack of methodologically rigorous and empirically strong evidence poses a challenge to drawing conclusions of a causal pathway between features of building design and health outcomes. For instance, it was not possible to rule out the role of residual confounding as a possible explanation for some of the findings due to the complexity of factors. Nevertheless our key findings are consistent with existing evidence and as such highlights the importance of policies and actions to promote the design of healthy and sustainable buildings.

### What is already known on this topic

Several reviews have reported an association between housing and health;^12,13^ albeit, many of these have adopted a narrow approach by examining the link between a specific feature of housing design, such as ventilation, and a certain aspect of health in a particular population, e.g. respiratory health among children. Identifying how several features of building design interact together and influence health and wellbeing across a life course can build a stronger case for informing collaborative actions.^51^ There is also insufficient review level evidence on the health outcomes and impact of non-residential building design.

### What this study adds

Unlike previous studies that only consider the association between elements of the building design and specific health outcomes, this review adopts a systematic and comprehensive approach to synthesize and assess the quality of all available evidence on the association between building design features and health at a population level. We have been able to identify important associations between early exposure to poor quality housing on health in later life, for instance, the links between housing conditions experienced in childhood and morbidity and mortality in later life have been discussed in detail.^36^

Our systematic approach of collating and assessing the quality of existing evidence has facilitated the identification of knowledge and research gaps in relation to the nature of evidence in this field and the need for more robust evidence investigating the associations between building design features and health. In particular, we report a substantial gap in the evidence base for non-residential buildings.

The need to promote health, wellbeing and safety in buildings through use of the evidence base has become an important policy focus globally.^52^ Indeed, the importance of developing building regulations that incorporates international guidance and evidence has been advocated by the World Health Organization.^53^ The WELL building standard was launched in 2014 to provide a global overview of best practices in design and construction that support health and wellbeing^54^ Several countries have developed standards for building design; for example building regulations in the UK now set minimum statutory standards for design, construction and alterations to nearly every building in the UK.^55,56^ However, building regulations do not necessarily consider the full evidence base linking building features and health impacts and, as minimum standards, often do not consider how building design could promote better health and improved wellbeing. The findings from our review provide evidence for those who are seeking to better integrate health and wellbeing considerations into building design.

### Limitations of the study

In most cases, the data collected from studies showed that several features of housing and health are related, but this relationship may not necessarily be causal. Some of the studies included in the review examined the impact of more than one housing intervention such as installation of central heating and improvements to ventilation. As such it was difficult to establish which of the interventions played a more substantial role in creating the observed health improvement. We were also not able to exclude the possibility of reverse causality of some of the reported associations. The association between housing and health could in fact be a reversible relationship where poor health can impact negatively on housing opportunities.^57,58^

The lack of detail of what constitutes inadequate housing and the lack of clarity of housing quality benchmarks used in some studies,^29,40,59^ suggests there was a reliance on value judgement by the researchers and professionals involved. This of course is highly subjective as the definition of adequate housing might be context specific. In the most part, the lack of evidence linking building design and health could be due both to the challenges associated with conducting experimental studies in the field and to the difficulty in capturing the impact of the wider social context.

### Conclusion

Findings from this study suggest that affordable housing of good quality, with good energy efficiency and adequate ventilation, has the potential to be an important contributor to improved health and wellbeing. The evidence detailed in this review can contribute to informing the development of health interventions and policy interventions, particularly with regard to the evaluation of existing standards and advancement of new standards in the built environment domain.

## Supplementary Material

Supplementary Data
