# Supplementary material for: The relationship between buildings and health: a systematic review
Source: J Public Health (Oxf). 2018 Aug 18;41(2):e121–32. doi: 10.1093/pubmed/fdy138 (PMC6645246; doi:10.1093/pubmed/fdy138)
Supplement: Supplementary Data [file fdy138_appendices.docx]

**Appendices**

| **Indicators for housing** | Building* OR office building OR shops OR school building OR Community buildings OR Leisure Facilities OR stadium OR malls OR religious buildings OR Tower OR warehouse OR church OR hotel OR factory OR club Apartment or ‘Better housing’ or Bungalow or Damp (home) or Dwelling Or ‘Healthy housing’ or Home* or Housing or ‘Independent living’ or ‘Living accommodation’ or ‘Neighbourhood renewal’ or Occupants or ‘Public housing’ or‘ recovery housing’ or Refurbish* NEAR/3 (home or houses) or Rehousing or Residential OR Rootlessness OR Shelter or Squatter or ‘supportive housing’ or Tenants or water supply NEAR/2 (hous*) or lead or noise or defensible space or insulation or ventilation or home adaptation or housing renewal or area renewal pathfinder or housing NEAR/ 1 (vulnerable) or private NEAR/1 (sector) or ‘council housing’ or social Near/1 (housing) or ‘housing association’ or ‘poor housing’ or ‘substandard housing’ or ‘condemned housing’ or mould NEAR/3 (hous*) or asbestos NEAR/3 (house) |
| --- | --- |
|  | AND |
| Health outcomes | Accident or asthma or ‘blood pressure’ or cardiovascular or COPD or CVD or death or disability or disorder or ‘emotional health’ or ‘health outcome’ or health or illness* or ‘injury prevent*’ or ‘life satisfaction’ or ‘medical condition*’ or ‘mental health’ or ‘Moderate-vigorous physical activity’ OR MVPA or obes* or physical activity or physical health or sedent* or suicide or violence or independence or mobility or safety or fall or depression or isolation or fire or ‘electrical hazards’ or wellbeing |
|  | AND |
| Study type | 'Experimental study' or intervention NEAR/2 (study) or RCT or 'before-and after' or case-control |

**Appendix 1: Search Strategy for MEDLINE**

**Appendix 2: Summary of included studies**

| **First author/Year** | **Country** | **Study design ^a^** | **Aim(s)** | **Feature of interest** | **Health outcome(s)** | **Participants** |
| --- | --- | --- | --- | --- | --- | --- |
| Barr, 2013 ^65^ | UK | Q | To evaluate the relative benefits of supportive accommodation | Provision of community step down housing for women in psychiatric care | General health | Women aged 18 to 67 years |
| Blackman, 2003 ^73^ | UK | L | To compare change in health status among adult applicants for medical priority housing who were rehoused with similar cohort who were not rehoused. | Relocation to medical priority housing | Health and wellbeing health | 18-92 years |
| Brohus, 2001 ^72^ | USA | Intervention study | To investigate the effect of renovating an office building on occupants’ comfort and health | Renovation of office building (installation of new heating, ventilation improvement and carpet fixing) | Comfort and health | Not specified- employees in an office building |
| Casciano, 2012 ^67^ | USA | Q | To test a hypothesis that living in an affordable housing project reduces a poor person’s exposure to disorder and violence and that reduced exposure to disorder and violence can lead to improvements in anxiety. | Affordable housing | Exposure to disorder, anxiety | Adults |
| Choi, 2014 ^30^ | South Korea | C-S | To determine the associations between building characteristics and children’s allergic symptoms in South Korea. | Location of home, type and size of residence, window type, floor covering, wall covering, heating system, ventilation system, renovation, balcony alteration, dampness and odours. | Wheeze, dry cough, rhinitis, asthma and eczema within previous 12 months | Children age 1-8 years |
| Doran, 2016 ^56^ | USA | B-A | To examine the characteristics of homeless people using emergency departments before and after Hurricane Sandy | Homelessness or inadequate housing after hurricane | Emergency department visit | Adults≥ 18 years |
| Fauth, 2004 ^59^ | USA | Q | To investigate the short-term effects of relocating from public houses in neighbourhood of higher poverty to neighbourhoods of lower poverty | Relocation to areas of lower poverty | General health effect | adults |
| Frisk, 2006 ^69^ | Sweden | Case-C | To compare housing condition of people with asthma and people who do not have asthma. | Type of ventilation, heating system, damp, mould, flooring | Asthma | 15-49 years |
| Gehring, 2008 ^27^ | Albania, Italy, New Zealand, Sweden, UK | C-S | To investigate the relationship between house dust exotoxin level and respiratory symptoms in children across several countries | House dust endotoxin level | Respiratory symptoms | Children age 9-12 years |
| Hamoudi 2013 ^66^ | USA | Q | To examine the health impact of the housing boom in United States between 1990’s and 2000’s. | Cost of housing | General health | Adults |
| Issa, 2011 ^70^ | Canada | Not specified | To investigate the impact of indoor environment of green schools on student’s performance and staff absenteeism | Indoor environment of green schools | Absenteeism, student performance and satisfaction | Students and teachers (age not specified) |
| Kelaher, 2010 ^68^ | Australia | B-A | To examine the impact of neighbourhood renewal (NR) on health and live satisfaction | Neighbourhood renewal | Health and life satisfaction | Adults>18 years |
| Lloyd, 2008 ^60^ | UK | B-A | To assess the impact of improving thermal quality of housing on blood pressure and general health | Housing improvement-thermal improvement | Changes in blood pressure, General health and financial status | Adults >18 years |
| Orrell, 2013 ^26^ | UK | C-S | To investigate the relationship between design of extra care houses and residents’ quality of life | Accessibility, safety and sensory support | Quality of life | Older adults >70 years |
| Oudin, 2015 ^28^ | Sweden | C-S | To examine the association between poor housing condition and health of children from disadvantaged immigrant population | Dampness, mould and presence of cockroaches) | General health | Children 0-13 years |
| Padro-Martinez ^61^ | USA | RCT | To examine whether filtration of particulate matter in public housing near a highway can reduce high blood pressure. | Air filtration intervention | Blood pressure | Children and adults |
| Rieke, 2015 ^62^ | USA | B-A | To examine the effect of supportive housing placement on hospital admissions of adults who were homeless. | Supportive housing programme | General health | Adults >19 years |
| Sharpe, 2015 ^29^ | UK | C-S | To assess how fuel poverty, occupants’ risk perception and use of mechanical ventilation mediate the risk of mould contamination in social housing. | Fuel poverty, ventilation | Mould contamination | Adults, mean age 60 |
| Shields, 2016 ^63^ | USA | Q | To quantify the unintentional injuries associated with housing elements among children below 18 years. | Bathtub, cabinet, carpet, ceiling/wall, counter, door, fence, floor, nail, porch, stairs and window | Unintentional injuries | Children <18 years |
| Shortt, 2005 ^64^ | UK | Q | To evaluate the effectiveness of a fuel poverty programme | Fuel poverty | Ill health | Children and adults |
| Somerville, 2000 ^58^ | UK | Q | To evaluate the impact of installing heating on health of children with asthma | Installation of heating | Asthma | Children <16 years |
| Tanner, 2008 ^71^ | USA | Not specified | To examine the effect of school design on student performance | School design (described as movement and circulation, day lighting ad views) | Academic performance | Fifth grade students |
| Thomas, 2005 ^57^ | UK | Case-C | To examine mental health consequences of single regeneration budget (SRB) | Single regeneration budget | Stress | General |
| Aylin, 2001 ^38^ | UK | E-S | To evaluate the associations between temperature, housing, deprivation and excess winter mortality | Housing, deprivation, temperature | Excess winter mortality | Adults ≥65 |
| Bentley, 2011 ^46^ | Australia | L | To investigate the effect of housing affordability on mental health among people with low household income. | Housing affordability | Mental health | 25-64 years |
| Blackman, 2001^41^ | UK | B-A | To investigate the association between neighbourhood renewal and health | Neighbourhood renewal (including fabric repairs and refurbishment) | Physical and mental health | Children and adults |
| Curl, 2015 ^32^ | UK | Q | To evaluate the impact of housing improvements on physical and mental health | Central heating, ‘secured by design’ front doors, fabric works, kitchens and bathrooms’) | Physical and mental health | Children and adults |
| Edward, 2011 ^34^ | UK | CEA nested in RCT | To evaluate the cost-effectiveness of installing ventilation systems in homes of children with moderate to severe asthma | Provision of adequate ventilation and heating | Incremental cost-efficiency ratio, Asthma | 5-14 years |
| Holtgrave, 2013 ^42^ | USA | CUA of RCT study | Cost utility analysis of the impact of provision of immediate rental housing assistance to people living with HIV/AIDS | Access to housing | HIV disease progression, medical care access and utilization, treatment adherence, mental and physical health, risk of transmitting HIV | ≥ 18 years |
| Howden-Chapman, 2008 ^32^ | New Zealand | RCT | To examine the effect of improved home heating on asthma among children. | Improved home heating (heat pump, wood pellet burner, flued gas) | Change in lung function, reported respiratory tract symptoms | Children aged 6-12 years |
| Kidder, 2007 ^43^ | USA | RCT | To assess the impact of provision of immediate rental housing assistance to people living with HIV/AIDS (PLWHA) who were homeless | Access to housing | HIV disease progression, medical care access and utilization, treatment adherence, mental and physical health, risk of transmitting HIV | ≥ 18 years |
| Leventhal, 2003 ^44^ | USA | RCT | To examine the short-term effects of relocation from public housing in neighbourhood of high poverty to private housing in low poverty areas on mental health | Relocation from public housing in neighbourhoods of high poverty to private housing in areas of lower poverty | Mental health, general wellbeing | Average age of parents=35  Average age of children= 12.62, SD=2.74 |
| Leventhal, 2004 ^45^ | USA | RCT | To investigate the impact of relocation to from public housing in neighbourhood of high poverty to private housing in low poverty areas on children’s achievement, grade retention, suspensions and expulsions | Relocation from neighbourhoods of high poverty to areas of lower poverty | Educational achievement, grade retention, suspension and expulsion | Average age of parents=35  Average age of children= 12.62, SD=2.74 |
| Vettore, 2010 ^40^ | Brazil | Case-C | To examine the relationship between housing condition and low birthweight and preterm low birthweight among low-income women. | Housing condition- described as adequate, inadequate and highly inadequate | Low birthweight, preterm low birth weight | Pregnant women aged ≥ 30 years and new born |
| Woodfine, 2011 ^33^ | UK | RCT | To evaluate the effectiveness of installing ventilation systems in the homes of children with moderate or severe asthma | Provision of adequate ventilation and heating | Asthma | 5-14 years |
| Barton, 2006 ^35^ | UK | RCT | To assess the short term (1 year period) health effects of housing improvement | Central heating, ventilation, rewiring, insulation and re-roofing) | General health effect | Children and adults |
| Dedman, 2001 ^36^ | UK | Cohort | To examine the association between measures of housing condition during childhood and all-cause mortality | Crowding, water supply, toilet facilities, adequacy of ventilation, cleanliness of households | All course mortality | Children |
| Howden-Chapman, 2007 ^31^ | New Zealand | Cluster RCT | To examine whether insulating existing houses can increase indoor temperature and improve occupants’ health and wellbeing. | Installation of standard retrofit insulation package | Indoor temperature, energy consumption, self-reported health, days off school or work, visit to hospital | General |
| Keall, 2015 ^39^ | New Zealand | Clustered RCT | To assess the safety benefit of home modifications | Handrails for step and stairs, grab rails for bathroom, outside lighting, edging for outside steps and slip-resistant surfacing for outside areas such as decks and porches | Falls, fall-related injury | Children and adults |

a: C-S= cross-sectional study, Q= quasi-experimental study, RCT=randomised controlled trial, L= longitudinal study, B-A= before-and-after study, CUA= cost utility analysis, Case-C= case control study, E-S= ecological study

**Appendix 3: Main findings from included studies**

| **First author, year of publication** | **Country** | **Main findings** | **Quality of study** |
| --- | --- | --- | --- |
| Barr, 2013 ^65^ | UK | Study to evaluate the relative benefits of supportive accommodation, comparing female patients in community step-down housing and a control group. Supportive accommodation led to improvement in psychological well-being P<0.05. | Low |
| Blackman, 2003 ^73^ | UK | Applicants for MPH were interviewed at the application stage 9 and 12 months follow up. Those granted the priority housing were grouped as ‘intervention’ while those who were placed on the waiting list were regarded as control group. The rehoused and not rehoused groups were not significantly different in terms of health status, gender, education, income or housing conditions, but participants who were not rehoused were more likely to report mobility problems and to be aged over 50. Those who were not rehoused experienced a slight improvement in five dimensions of the SF‐36 whereas those who were rehoused experienced much larger improvements in six dimensions. For those who were rehoused, significant net improvements occurred in reports of tiredness, feeling depressed, sleeplessness, use of prescribed medication, use of medical services and problems with IADL. | Low |
| Brohus, 2001 ^72^ | USA | A comprehensive indoor climate investigation was performed before and after the intervention. Occupants completed a daily questionnaire regarding their comfort and health. Physiological examinations of eyes, nose and lungs was also performed. The authors reported that the intervention significantly reduced the severity of occupants’ environmental perceptions and symptoms. | Low |
| Casciano, 2012 ^67^ | USA | Findings showed that residents living in affordable housing project in a middle-class suburb were less likely to be exposed to disorder and violence and had lower stress levels and slightly fewer anxiety symptoms. Non-residents mean weighted disorder score (i.e. total exposure to disorder) was almost 6 times higher than that of the residents (54.6 and 9.3 respectively) t=7.652, p=0.000. | Low |
| Choi, 2014 ^30^ | South Korea | After controlling for some potential confounders (gender, age, family allergy, socioeconomic status, environmental tobacco smoke), the result showed that PVC flooring (AOR=1.54; 95%CI=1.13-2.09) and Linoleum flooring (AOR=1.58, 95%CI=1.14-2.20) were associated with eczema in the previous 6 months. Floor moisture also significantly increased the association between PVC and symptoms of wheezing (AOR=2.57; 95%CI=1.36-4.82) and eczema (AOR=1.97, 95%CI=1.18-3.28).  The cross-sectional nature of the study implies that casual links cannot be inferred. | Low |
| Doran, 2016 ^56^ | USA | There was a statistically significant increase in ED visits for homeless people (or those living in inadequate housing) in the week after Hurricane Sandy’s landfall. Those accessing the ED for homelessness or inadequate housing were more often elderly. | Low |
| Fauth, 2004 ^59^ | USA | Adults who moved to neighbourhoods of lower poverty were less likely to: experience victimisation (-0.19 Standard error=0.07, P<0.001), abuse alcohol (-0.72 standard error=0.39, P<0.10) and were more likely to report satisfaction with neighbourhood resources (0.13 standard error=0.07, P<0.10) when compared with counterparts who remained in areas of high poverty. However, adults who moved to neighbourhoods of lower poverty were less likely to socialize informally with neighbours than those who stayed in neighbourhoods of higher poverty (-0.71 standard error=0.13 p<0.01) | Low |
| Frisk, 2006 ^69^ | Sweden | Result shows no significant difference in the housing environments between the two groups. The only exception was in terms of mean CO_2_ level of empty house. Controls (610) had a higher mean CO_2_ level than cases (680) p=0.041. | Low |
| Gehring, 2008 ^27^ | Albania, Italy, New Zealand, Sweden, UK | Data was retrieved from an International study in 5 countries (Albania, Italy, New Zealand, Sweden and United Kingdom). Results combined across all countries showed that endotoxin level was inversely associated with whether or not children ever had asthma (adjusted OR=0.53 95% CI= 0.29-0.96). | Low |
| Hamoudi 2013 ^66^ | USA | Increase in housing wealth was associated with better health outcomes for homeowners in late middle-age and older. Respondents living in communities where value of housing was higher had fewer functional limitations (such as reduced mobility) and lower waist circumference compared to neighbourhoods where price of housing was stagnant. Difficulties with activities of daily living among homeowners were reported b (SE) = -6.3 (3.2) P=0.05. | Low |
| Issa, 2011 ^70^ | Canada | Student teachers and staff absenteeism in green schools improved by 2-7% while student performance improved by 8-19%. However, these associations were not statistically significant. Teachers in green schools and energy retrofitted schools were less satisfied with noise acoustics than teachers in convectional schools (P=0.012 and 0.024 respectively).  Small sample size (150 teacher sampled). Only 3 green schools were sampled while 20 retrofitted schools were included. | Low |
| Kelaher, 2010 ^68^ | Australia | Study to examine the impact of neighbourhood renewal (NR) on health and live satisfaction. NR improved health (OR=2.15 95%CI=1.23-3.74) and life satisfaction (OR=1.79 95%CI= 1.38-1.52) of residents compared to people living in other areas. | Low |
| Lloyd, 2008 ^60^ | UK | Systolic and diastolic blood pressure fell significantly (P<0.000) in intervention group. Improvement in general health was reported (self-reported) and indicated by reduction in use of medication and hospital admission. There was also reduction in heating costs and other expenses in the intervention group. The control group did not experience any change in outcome measurement. | Low |
| Orrell, 2013 ^26^ | UK | A stratified sample of 35 extra care housing schemes from private and public sector were sampled (23 schemes agreed to participate). Quality of life was accessed using the SEIQoL-DW.  Several aspects of building design were found to affect the quality of life of residents. Elements of the design relating to safety, accessibility and working care were negatively associated with resident’s quality of life while security was positively associated with resident’s quality of life. Building design was mediated by dependency which had a negative impact of QoL  This study had a small sample size (N=163), participants were self-selected by scheme managers, hence results cannot be generalised. | Low |
| Oudin, 2015 ^28^ | Sweden | Housing conditions in the study sites were generally poor. 67% of the apartments in one neighbourhood had been sanitised of cockroaches while 275 where infested with cockroaches and 40% had visible mould. There was a statistically significant association between current asthma and dampness (adjusted OR=2.8, 95%CI=1.2-6.4) and between mould and headache (adjusted OR=4.2, 95% CI= 1.2-14.8). Presence of cockroaches was associated with ≥2 visit to emergency care unit (adjusted OR=4.88, 95%CI=1.63-14.63). Presence of cockroaches was also associated with colds (adjusted OR=2.87, 95%CI=1.01-8.18), headache (adjusted OR=5.16, 95%CI=1.93-13.92) and sleep disorder (adjusted OR=8.61 95%CI=2.52-29.43). | Low |
| Padro-Martinez ^61^ | USA | There was no significant positive association between the intervention (high-efficiency particulate arrestance) and blood pressure. The intervention showed negative associations between certain biomarkers of blood pressure. The authors noted that these negative associations could be a result of confounding factors that were unaccounted for or errors in design of trial such as unsuccessful randomisation. | Low |
| Rieke, 2015 ^62^ | USA | Results show a reduction in the number of admissions to emergency department and an increase in outpatient visits, suggesting that supportive housing may encourage more appropriate use of health care services. | Low |
| Sharpe, 2015 ^29^ | UK | Participants living with inadequate heating or those unable to heat their homes due to cost has an increased risk of mould contamination (OR=3.495% CI=2.0-5.8 and OR=2.295% CI=1.5-3.2 respectively). | Low |
| Shields, 2016 ^63^ | USA | Estimates of emergency department visits associated with identified housing elements ranged from 6.34 per 100,000 annually for injuries associated with counters to 69.96 per 100,000 annually for injuries associated with floors. Stairs, bathtubs, ceilings and protruding nails resulted in 49.33, 16.37 25.39 and 10.27 (per 100,000 annually) respectively. | Low |
| Shortt, 2005 ^64^ | UK | The intervention led to improvements in energy efficiency, health and wellbeing and also increase comfort levels in the home. Prior to the intervention, 76% of households reported presence of condensation, mould and damp (CMD). However, after installation of central heating 60% (of the76%) reported that CMD were no longer present. P<0.001. | Low |
| Somerville, 2000 ^58^ | UK | Following the intervention (installation of gas central heating, electric storage heaters, solid-fuel central heating and oil-fired central heating), all respiratory symptoms were significantly reduced. Nocturnal cough reduced from a median score of 3 to 1 (p<0.001) in the previous month. School age children lost significantly less time from school for asthma in the previous 3 months (9.3days per 100 days before the intervention to 2.1 days afterwards. P<0.001).  The sample size for this study was small (104 at baseline and 72 at 3 months follow up). | Low |
| Tanner, 2008 ^71^ | USA | Movement and circulation comprised of features such as outside walkways, pathways, point of reference and connectivity of buildings and outdoor spaces. Patterns of views comprised the following features: views overlooking life, unrestricted views, living views, functional views and green areas. Based on the three key areas of investigation, a questionnaire was designed to be completed by the students. Their responses were correlated with academic performance. Result shows that the design of schools has a significant effect on reading vocabulary, reading comprehension, language, arts, mathematics and science. | Low |
| Thomas, 2005 ^57^ | UK | Stress was higher in the study group (group that received SRB) (GQH12 score=2.621) compared to the matched control group (GQH12 score=2.307) p=0.019. This finding could have been due to the additional environmental nuisance they encountered. | Low |
| Aylin, 2001 ^38^ | UK | There was a significant association between excess winter mortality and temperature. For every 1^0^C reduction in 24hours mean winter temperature there was a 1.5% increased odd of dying. Associations between housing and winter mortality were not statistically significant; however, lack of central heating was associated with higher risk of dying in winter (OR= 1.016, 95% CI=1.009-1.022). | Moderate |
| Bentley, 2011 ^46^ | Australia | This study was performed to evaluate the association between living in a house where the housing cost was more than 30% of household income and mental health. Data for the study was retrieved from an Australian National longitudinal survey. Mental health was measured using the self-completed Short Form SF-36 measure. The authors found that entering unaffordable housing for individuals living in low-to-moderate income households was associated with a slight decrease in mental health score (mean change =-1.19, 96%CI= -1.97 to -0.41). There was no evidence for an association between mental health and affordable housing for higher income earners. | Moderate |
| Blackman, 2001^41^ | UK | After controlling for confounding variables, findings showed that an adult living in a damp house is significantly more likely to have one or more acute respiratory condition (OR=2.1 95%CI=1.26-3.5). Findings also show that perception of an area as being unsafe can increase odds of mental health problems (adjusted OR=2.35 95%CI=1.41-3.92). An adult living in in a dwelling with serious drought is significantly more likely to report a mental health problem than one living in a dwelling with minor or no drought (OR=2.28 95%CI=1.41-3.69).  In terms of children, living in a damp house increases odds of one or more respiratory problems by 3.5 (95%CI= 1.69-7.18). | Moderate |
| Curl, 2015 ^32^ | UK | Fabric works (which includes over-cladding and insulation) showed positive associations with physical health (+2.09, 95% CI 0.13 to 4.04) and mental health (+1.84, 95% CI 0.04 to 3.65) in 1–2 years. Improvements to kitchens and bathrooms demonstrated a positive association with mental health in 1–2 years (+2.58, 95% CI 0.79 to 4.36). Central heating had a negative association with physical health (−2.21, 95% CI −3.74 to −0.68). New front doors had a positive association with mental health in <1 year (+5.89, 95% CI 0.65 to 11.14) and when provided alongside kitchens and bathrooms (+4.25, 95% CI 1.71 to 6.80). | Moderate |
| Edward, 2011 ^34^ | UK | The intervention (described in Woodfine, 2011) was successful in shifting 17% of children with severe asthma to moderate asthma, compared with a 3% shift in the control group. The mean cost of the intervention was £1718 per child treated or £12,300 per child shifted from severe to moderate. An incremental cost efficiency ratio (ICER) of £234 was obtained per point improvement on the 100-point asthma scale (PedsQL). 95% confidence interval (CI) = £140 to £590. ICER declined to £165 (95%CI = £84 to £424) for children with ‘severe’ asthma. | Moderate |
| Holtgrave, 2013 ^42^ | USA | Cost Utility Analysis based on findings from a randomised controlled study to examine the impact of provision of immediate rental housing assistance to people living with HIV/AIDS (Housing and Health study described in Kidder, 2007). The cost per QALY saved by provision of rental housing assistance to homeless PLWHA was $62,493. | Moderate |
| Howden-Chapman, 2008 ^32^ | New Zealand | The intervention group were provided with non-polluting, more effective home heating before winter. The control group received replacement heater at the end of the trial. There was no significant difference in improvement in lung function among intervention and control group at the end of 1 year.  However, children in the intervention group had 1.80 fewer days off school (95%CI=0.11-3.13), 0.40 fewer visits to doctor for asthma (95% CI=0.11-0.62), and 0.25 fewer visits to a pharmacist for asthma (0.09 to 0.32). Children in the intervention group also had fewer reports of poor health (adjusted odds ratio 0.48, 95% confidence interval 0.31 - 0.74), less sleep disturbed by wheezing (0.55, 0.35 - 0.85), less dry cough at night (0.52, 0.32 to 0.83), and reduced scores for lower respiratory tract symptoms (0.77, 0.73 to 0.81) than children in the control group.  The intervention was associated with a mean temperature rise in the living room of 1.10°C(95%confidence interval 0.54° C to 1.64°C) and in the child’s bedroom of 0.57°C (0.05°C to 1.08°C). Lower levels of nitrogen dioxide were measured in the living rooms of the intervention households than in those of the control households (geometric mean 8.5 μg/m3v 15.7 μg/m3, P<0.001). A similar effect was found in the children’s bedrooms (7.3 μg/m3v 10.9 μg/m3, P<0.001). | Moderate |
| Kidder, 2007 ^43^ | USA | 630 participants completed baseline assessment and were randomised to either receive immediate rental housing assistance (intervention) or assistance with finding housing according to standard practice (control). Findings demonstrate that health status of homeless people was poorer than that of housed respondents. Homeless respondents were also more likely to have visited an emergency department, and to have been admitted to a hospital. 40% of homeless respondents (compared to 26% of housed respondents) were more likely to have visited emergency department (P<0.001, X^2^= 32.2). Relative to 21% of housed respondents, 37% of homeless participants were more likely to have been admitted in the hospital in the past 12 months (P<0.001, X^2^= 42.3). Homeless respondents had lower CD4 counts, were less likely to adhere to anti-retroviral therapy. | Moderate |
| Leventhal, 2003 ^44^ | USA | Parents who moved to areas of low- poverty reported significantly less distress than counterparts who remained in areas of high poverty. Young boys who relocated to areas of lower poverty also reported significantly fewer anxiety issues than mates in public housing. There was a 20% reduction in depressive symptoms among experimental parents than control parents (P<0.001) | Moderate |
| Leventhal, 2004 ^45^ | USA | Data from Leventhal 2003 was examined to assess whether moving from high poverty neighbourhoods to low poverty areas was associated with low-income minority children’s achievement, grade retention, suspensions and expulsions. Findings showed that moving to low-poverty neighbourhoods had a positive effect on 11–18-year-old boys’ achievement scores compared with those of their peers in high-poverty neighbourhoods. | Moderate |
| Vettore, 2010 ^40^ | Brazil | Housing conditions were grouped into three categories: adequate, inadequate and highly inadequate. Findings showed that poor housing conditions was independently associated with low birthweight (inadequate-adjusted OR 2.2 C.I-=1.1-4.3 highly inadequate-adjusted OR=7.6 CI=2.4-23.9). | Moderate |
| Woodfine, 2011 ^33^ | UK | The intervention improved parent-reported asthma specific quality of life significantly at both 4 and 12months. The adjusted mean difference for the PedsQL asthma summary score of the two groups at 12months was 7.1 points (95%confidence interval [CI] = 2.8 to 11.4, P = 0.001; standardised effect size= 0.42). The generic quality-of-life scale showed that health problems were significantly reduced at 4months (adjusted mean difference of 7.2 (95%CI = 2.6 to 11.8, P = 0.002), while result were not significant at 12months (mean difference= 4.5, 95%CI = –0.2 to 9.1, P = 0.061). School attendance was higher in the intervention than the control group but not significantly (Mann–Whitney U tests: P = 0.091 for all-cause absence, P = 0.053 for asthma-related absence). | Moderate |
| Barton, 2006 ^35^ | UK | Houses and residents were randomised to two groups: group 1 received upgrade to ventilation heating, insulation and other home improvements in the first year (intervention group) and group 2 received the same kind of upgrade in the second year (control group). A postal questionnaire was sent to residents; outcomes were measured using annual health questionnaires SF36 and GHQ12. All adults were later interviewed by a trained community nurse.  The interventions (central heating, ventilation, rewiring, insulation and re-roofing) improved energy efficiency. Residents of un-improved houses (control group) reported increase in non-asthma-related chest problems including bronchitis, dry throat, itchy eyes, blocked nose and runny nose (Mann-Whitney test, z=2.8 p=0.05). Adults in improved houses showed improvement in combined asthma symptom score (Mann-Whitney test, Z=2.7; p=0.07). There was no difference between intervention and control was observed for SF36 or GHQ12 (tools for measuring general health). | High |
| Dedman, 2001 ^31^ | UK | Inadequate housing conditions were generally associated with increased adult mortality. After adjusting for childhood and adult socioeconomic factors, private indoor tapped water supply was significantly associated with increased mortality from coronary heart disease (hazard ratio 1.73, (95% CI 1.13, 2.64); Similarly, a significant association was observed between poor ventilation and overall mortality (hazard ratio for people from households with poorest ventilation relative to best ventilation 1.30, 95% CI 0.97, 1.74). | High |
| Howden-Chapman, 2007 ^31^ | New Zealand | The intervention group reported a slight increase in bedroom temperatures during the winter (0.5°C) and decrease in relative humidity (−2.3%). However, energy consumption in insulated houses was 81% that of uninsulated houses. These changes were significantly associated with reduced odds of fair or poor self-rated health (adjusted odds ratio=0.50, 95% confidence interval =0.38 to 0.68), self-reports of wheezing in the past three months (OR=0.57, 0.47 to 0.70), self-reports of children taking a day off school (0.49, 0.31 to 0.80), and self-reports of adults taking a day off work (0.62, 0.46 to 0.83). The odds of hospital visits were lower among occupants of insulated homes (0.73, 0.62 to 0.87). Hospital admissions for respiratory conditions were also reduced (0.53, 0.22 to 1.29), but this reduction was not statistically significant (P=0.16). | High |
| Keall, 2015 ^39^ | New Zealand | Households were randomly assigned into immediate home- modification (intervention group) or the three year wait before modification (control group). Findings show that following 1148 days of randomisation, the crude rate of fall injuries per person per year in the intervention and control group was 0.061 and 0.072 respectively.  In addition, the crude rate of injuries specific to home modification intervention was 0.018 in the intervention group and 0.028 in the control group. After adjusting for relevant confounders, there was a 26% reduction in the rate of home injuries caused by falls in the group that received home modification (relative risk=0.74 CI=0.058-0.94). Injuries specific to the intervention also declined by 39% per year among those that received the intervention (RR=0.61, CI=0.41-0.91) | High |
